# Genetic and Pathogenic Characteristics of Novel PRRSV‐1 Strain CH‐JX‐2504 and CH‐SDTA‐2506 in China

**DOI:** 10.1155/tbed/4674029

**Published:** 2026-08-24

**Authors:** Yulin Xu, Qihang Xin, Kexin Li, Jianda Li, Luogang Ding, Yuyu Zhang, Hao Zeng, Fei Liu, Zhi Chen, Wenbo Sun, Jiang Yu, Jiaqiang Wu

**Affiliations:** ^1^ Key Laboratory of Livestock and Poultry Multi-Omics of Ministry of Agriculture and Rural Affairs (MARA), Institute of Animal Science and Veterinary Medicine, Shandong Academy of Agricultural Sciences, Jinan, China, saas.ac.cn; ^2^ School of Life Sciences, Shandong Normal University, Jinan, China, sdnu.edu.cn

**Keywords:** deletion patterns, isolation, metagenomic analysis, pathogenicity, porcine reproductive and respiratory syndrome virus 1

## Abstract

Porcine reproductive and respiratory syndrome virus (PRRSV) remains a leading cause of severe economic losses in the worldwide swine industry. In recent years, the geographic distribution of PRRSV‐1 has been expanding, further complicating epidemic prevention and control. In 2025, two PRRSV‐1 strains were successfully isolated from lung tissue samples of deceased pigs in Jiangxi and Shandong Provinces of China, named CH‐JX‐2504 and CH‐SDTA‐2506, respectively. Viral isolation was performed using primary porcine alveolar macrophages, and whole‐genome sequencing was performed through metagenomic analysis. Subsequent phylogenetic analysis indicated that strain CH‐JX‐2504 belonged to the new subgroup 3, while CH‐SDTA‐2506 clustered within the BJEU06‐1‐like subgroup. Amino acid sequence analysis showed that CH‐JX‐2504 exhibits identical deletion patterns in the Nsp2, GP3, and GP4 proteins with the PRRSV‐1 reference strain 180900‐5. In contrast, distinct variations in these three proteins were identified in CH‐SDTA‐2506 compared with other representative PRRSV‐1 strains, suggesting the emergence of a novel deletion pattern. In vivo challenge experiments showed that both CH‐JX‐2504 and CH‐SDTA‐2506 could induce typical clinical symptoms in piglets, including fever, retarded weight gain, and pathological lesions, including interstitial pneumonia with lymphocyte infiltration, obvious damage to intestinal villi, and disruption of the intestinal microbiota structure. Notably, one piglet in the CH‐JX‐2504 group died at 10 days postinfection (dpi), indicating that CH‐JX‐2504 exhibits higher pathogenicity than CH‐SDTA‐2506. Therefore, strengthened surveillance of PRRSV‐1 in China is essential to prevent its further spread.

## 1. Introduction

Porcine reproductive and respiratory syndrome (PRRS) remains one of the most devastating diseases in swine, causing tremendous economic losses to the global swine industry [1]. PRRS was first discovered in the United States in 1987 and subsequently reported in central Europe [2]. Porcine reproductive and respiratory syndrome virus (PRRSV), the pathogen that causes PRRS, is an enveloped and positive single‐stranded RNA virus belonging to the family Arteriviridae [3]. The PRRSV genome is ~15 kb in length and encodes at least 10 open reading frames (ORFs). ORF1a and ORF1b account for ~80% of the viral genome and encode the polyproteins pp1a and pp1ab, respectively. These polyproteins are proteolytically cleaved into 16 nonstructural proteins (Nsps) essential for viral replication and transcription, including Nsp1α, Nsp1β, Nsp2, Nsp2TF, Nsp2N, Nsp3‐6, Nsp7α, Nsp7β, and Nsp8‐12. In contrast, ORF2‐7 encodes eight structural proteins that assemble the viral particle, namely, GP2a, GP3, GP4, GP5, E, ORF5a, M, and N [3, 4].

PRRSV exhibits remarkable genetic and antigenic variability, which divides it into two species: *Betaarterivirus suid 1* (PRRSV‐1) and *Betaarterivirus suid* 2 (PRRSV‐2) [5], sharing 50–70% genetic similarity [6]. Global PRRSV‐2 can be classified into 11 distinct lineages, whereas PRRSV‐1 is divided into four lineages (L1–L4) [1, 7]. From the perspective of global epidemic geographical characteristics, PRRSV‐2 is predominantly prevalent in North America and Asia [7], while PRRSV‐1 mainly circulates in European countries. In China, PRRSV‐1 was first detected in 1997 [8]. Subsequently, Chen et al. [9] successfully isolated the BJEU06 and NMEU09 strains in China for the first time in 2011. Currently, all PRRSV‐1 isolates in China belong to lineage 1, and both their prevalence and virulence have shown a gradual increasing trend [10–12]. Meanwhile, the recombination mechanisms of Chinese PRRSV‐1 strains are highly intricate, with high‐frequency recombination sites mainly located in the Nsp2 and the GP2–GP4 regions [13, 14].

Previous studies have confirmed the prevalence of PRRSV‐1 in at least 28 provinces and regions across China [13], but the pathogenic features of novel PRRSV‐1 isolates remain very limited. In 2025, we collected samples from the lung tissues of one deceased pig each in Jiangxi and Shandong Provinces, and successfully isolated two PRRSV‐1 strains. We comprehensively analyzed their genomic characteristics and genetic evolutionary relationships and further evaluated their pathogenicity in piglets.

## 2. Materials and Methods

### 2.1. Virus Isolation

Lung tissue samples collected from one deceased pig each in Jiangxi and Shandong Provinces tested positive for PRRSV‐1 via real‐time RT‐PCR. Before virus inoculation, the lung samples were homogenized in PBS, and the homogenates were filtered through 0.22 µm filters. The filtrates were then inoculated into primary alveolar macrophages (PAMs). The cells were cultured in RPMI‐1640 medium supplemented with 2% fetal bovine serum (FBS) and antibiotics (100 U/mL of penicillin and 100 μg/mL streptomycin) at 37°C under a 5% CO_2_ atmosphere. Cell cultures were harvested at 3 days post‐inoculation and stored at −80°C for viral stocks.

### 2.2. Whole Genome Sequencing of PRRSV Isolate

Total RNA was extracted from the samples using the Simply P Viral RNA Extraction Kit (BioFlux, Beijing, China) according to the manufacturer’s instructions. Subsequently, cDNA was synthesized using the HiScript III RT Super Mix for real‐time PCR (qPCR) (+DNA wiper) (Vazyme, Nanjing, China) following the manufacturer’s protocol and sent to Shanghai Tanpu Biotechnology Co., Ltd. (Shanghai, China) for metagenomic sequencing.

### 2.3. Phylogenetic, Recombination, and Amino Acid Mutation Analysis

To elucidate the phylogenetic relationships among various PRRSV‐1 strains prevalent in China. The complete genome sequence of CH‐JX‐2504 and CH‐SDTA‐2506 were compared with 90 PRRSV reference strains retrieved from GenBank. Phylogenetic trees based on the complete genome and ORF5 gene were constructed using the neighbor‐joining method in MEGA 7.0 [15]. Bootstrap analysis with 1000 replicates was performed to assess the reliability of the phylogenetic tree topologies [16]. The full genomic sequences of CH‐JX‐2504, CH‐SDTA‐2506, and other PRRSV‐1 strains were subjected to recombination analysis using RDP4 and SimPlot 3.5.1 software. Seven algorithms integrated in RDP4 software, including RDP, GENECONV, BootScran, MaxChi, Chimaera, SiScan, and 3Seq, were applied to identify potential recombination events and corresponding breakpoints [17]. Further similarity comparisons were performed using SimPlot 3.5.1 within a 200 bp sliding window along the genome ratio (20 bp steps). Additionally, amino acid mutation characteristics of GP3, GP4, and Nsp2 proteins were analyzed by sequence alignment using the Clustal W module embedded in DNASTAR software.

### 2.4. Animal Challenge

To evaluate the pathogenicity of the novel PRRSV‐1 isolates CH‐JX‐2504 and CH‐SDTA‐2506, an animal challenge experiment was conducted using 5‐week‐old PRRSV‐free piglets. Thirteen piglets were randomly divided into three groups: the CH‐JX‐2504 inoculation group (*n* = 5), the CH‐SDTA‐2506 inoculation group (*n* = 5), and the control group (*n* = 3). Piglets in the two challenge groups were intranasally and intramuscularly inoculated with 2 mL of 10^5.0^TCID_50_/mL CH‐JX‐2504 or CH‐SDTA‐2506, respectively. Piglets in the control group were inoculated with RPMI‐1640 medium as a negative‐infection group. Rectal temperatures were recorded daily after inoculation, and clinical symptoms, including appetite, mental state, and respiratory manifestations, were continuously monitored. Body weights were measured weekly, and serum samples were collected periodically to detect viremia and PRRSV‐specific antibody levels. Serum PRRSV antibodies were detected using the VDPro PRRSV AB ELISA kit (WanBo, Beijing, China), with a sample‐to‐positive (s/p) ratio of 0.4 defined as the seroconversion threshold. All surviving piglets were euthanized at 21 days post‐inoculation, and lung tissue samples were harvested for subsequent histopathological examination.

### 2.5. Metagenomic Sequencing Analysis

Genomic DNA was extracted from jejunal contents using the Microbiome DNA Kit (Magen, Guangzhou, China) following the manufacturer’s protocol. DNA quality was detected using Qubit (Thermo Fisher Scientific, Waltham, MA) and Nanodrop (Thermo Fisher Scientific, Waltham, MA) accordingly. Qualified genomic DNA was fragmented to 350 bp by sonication, followed by end‐repaired, A‐tailed, and adaptor ligation with the NEBNext MLtraTM DNA Library Prep Kit for Illumina (NEB, USA) according to the protocol. Constructed libraries were purified with AMPure XP system (Beckman Coulter, Brea, CA, USA). Library fragment size distribution was verified on an Agilent 2100 Bioanalyzer (Agilent Technologies, Germany), and library quantification was performed using qPCR. Paired‐end 150 bp (PE150) sequencing was conducted on the NovaSeq X Plus platform (Illumina, San Diego, CA, USA). Raw sequencing reads generated from the Illumina platform were quality‐filtered with FASTP 0.20.0. Clean reads from each sample were assembled using MEGAHIT 1.2.9, and gene prediction was implemented with MetaGeneMark 3.38. All bioinformatic analyses were performed in R software. Statistical significance was defined as *p* < 0.05.

### 2.6. Statistical Analyses

Data represent the mean ± SD from three independent repeated experiments. Statistical analyses were performed using GraphPad Prism 8.0. Paired two‐tailed *t*‐tests were used, and a *p* < 0.05 was defined as statistically significant.

## 3. Results

### 3.1. Virus Isolation

**Figure 1:**
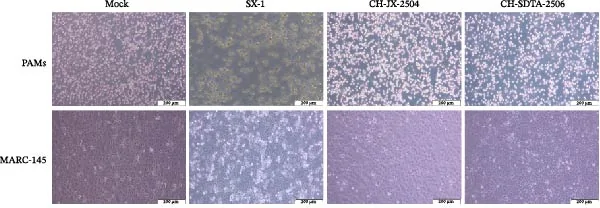
CH‐JX‐2504 and CH‐SDTA‐2506 were successfully isolated from PAMs. MARC‐145 cells and PAMs showed characteristic lesions. HP‐PRRSV‐2 SX‐1 isolate was used as a positive control for MARC‐145 cell infection.

**Figure 2:**
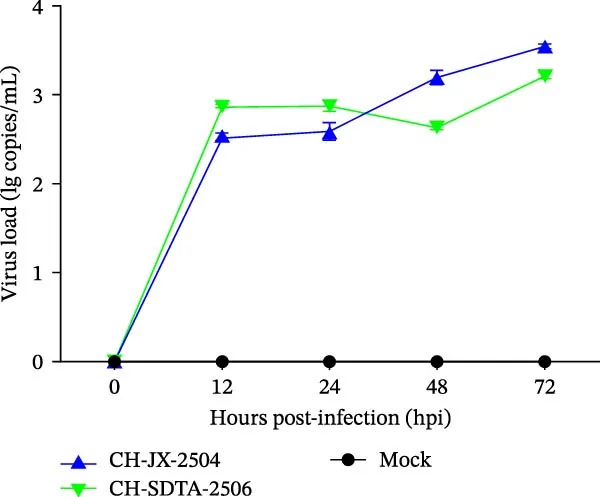
Growth kinetics of CH‐JX‐2504 and CH‐SDTA‐2506. The growth curves showed that the replication levels of the two isolates increased gradually with infection time and reached a peak at 72 hpi.

Two PRRSV‐1 strains were successfully isolated from pig farms in Jiangxi and Shandong Provinces and designated CH‐JX‐2504 and CH‐SDTA‐2506, respectively. The filtered tissue homogenates were inoculated onto PAMs and MARC‐145 cells. After three blind passages, no cytopathic effect (CPE) was observed in MARC‐145 cells, suggesting that neither CH‐JX‐2504 nor CH‐SDTA‐2506 was capable of replicating in this cell line. In contrast, significant cytopathology was observed in PAMs infected with both isolates at 72 h (Figure 1). Furthermore, the growth kinetics of CH‐JX‐2504 and CH‐SDTA‐2506 were assessed in PAMs. The results demonstrated that the replication levels of both isolates increased gradually with the extension of infection time and peaked at 72 hpi (Figure 2), which confirmed the successful isolation of CH‐JX‐2504 and CH‐SDTA‐2506 in PAMs.

### 3.2. Metagenomic Sequencing‐Based Analysis of the Complete Genome of PRRSV‐1

Full‐length genomes of isolates CH‐JX‐2504 and CH‐SDTA‐2506 were obtained by metagenomic sequencing. For CH‐JX‐2504, 7.48 million 150 bp paired‐end clean reads were generated. Quality tests showed that the Q20 and Q30 rates were 98.30% and 92.66%, respectively (Supporting Information 1: Table S1). For CH‐SDTA‐2506, 9.59 million 150 bp paired‐end clean reads were acquired, with Q20 and Q30 rates of 98.72% and 94.24%, respectively (Supporting Information 2: Table S2). These results indicated the satisfactory data quality. After filtering ribosomal RNA, host contamination, and bacterial sequences, 0.25 million reads (6.72%) from CH‐JX‐2504 and 0.073 million reads (1.53%) from CH‐SDTA‐2506 were obtained for virus mapping. Based on the above valid data, the complete genome sequences of both isolates were successfully assembled. The genome length of CH‐JX‐2504 was 15056 bp (Figure 3A), and the average coverage for the entire sequence was 1609.53 (Figure 3B), while the genome length of CH‐SDTA‐2506 was 15112 bp (Figure 3C), and the average coverage for the entire sequence was 36.84 (Figure 3D). BLAST analysis revealed that CH‐JX‐2504 shared the highest similarity (95.5%) with PRRSV‐1 180900‐5 isolate (MK303390.1), whereas CH‐SDTA‐2506 exhibited the highest similarity (only 87.1%) with PRRSV‐1 HeB3 (MN927227.1).

Figure 3Metagenomic sequencing determined the genomes of the PRRSV‐1 strains CH‐JX‐2504 and CH‐SDTA‐2506. (A) A 15056 bp sequence of CH‐JX‐2504 was obtained via metagenomic sequencing. (B) The average coverage of the CH‐JX‐2504 genome is 1609.53, and this strain shared the highest similarity with the PRRSV‐1 180900‐5 isolate. (C) A 15112 bp sequence of CH‐SDTA‐2506 was obtained via metagenomic sequencing. (D) The average coverage of the CH‐SDTA‐2506 genome is 36.84, and this strain shared the highest similarity with the PRRSV‐1 HeB3 isolate.

**Figure:**
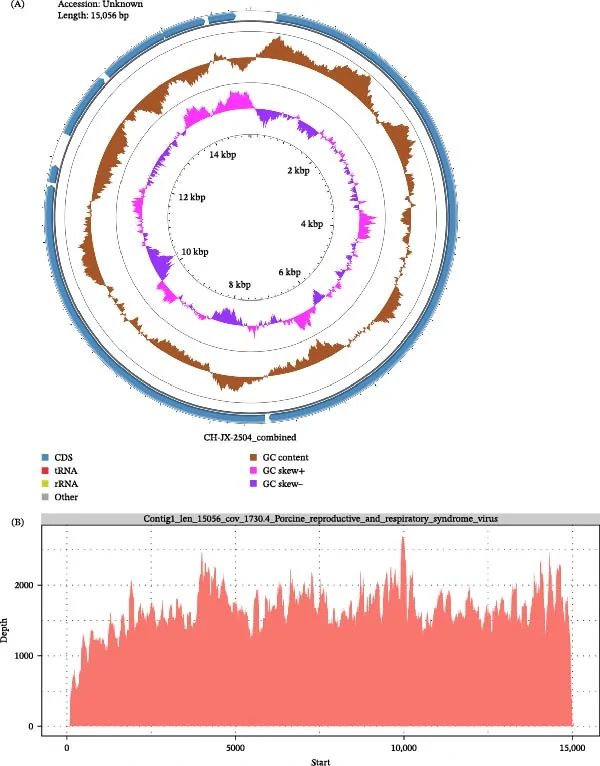

**Figure:**
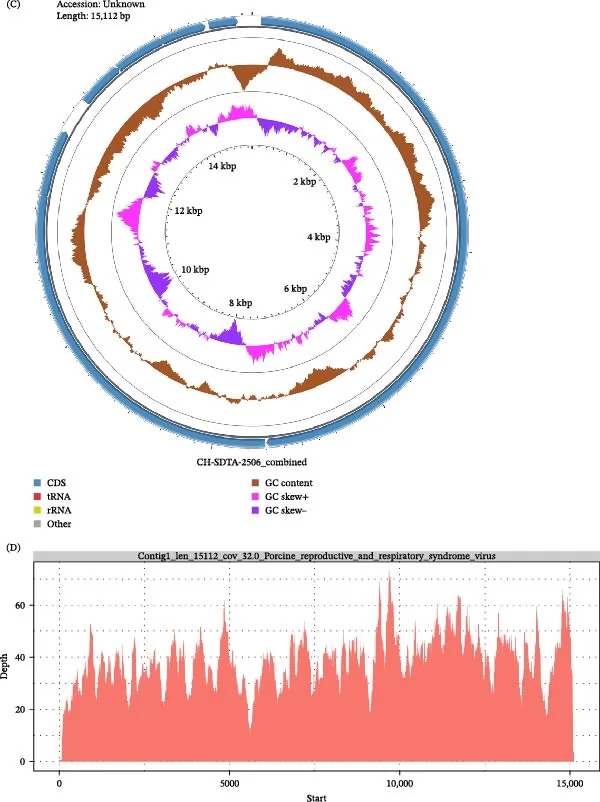

To clarify the phylogenetic relationships of CH‐JX‐2504 and CH‐SDTA‐2506 with prevalent Chinese PRRSV‐1 strains, phylogenetic trees were constructed based on the complete genome and ORF5 gene sequences. The CH‐JX‐2504 isolate clustered into the new subgroup 3 (L1.17) in both the whole‐genome and ORF5 phylogenetic trees (Figure 4A,B). In contrast, the CH‐SDTA‐2506 isolate was classified into the BJEU06‐1‐like subgroup (L1.13) in both phylogenetic trees (Figure 4A,B). Recombination analysis by RDP4 and SimPlot software suggested that neither CH‐JX‐2504 nor CH‐SDTA‐2506 exhibited recombinant signatures (data not shown).

Figure 4Phylogenetic analysis of 90 representative PRRSV strains based on the whole genome (A) and ORF5 gene sequences (B). CH‐JX‐2504 isolates clustered with the new subgroup 3 isolated and CH‐SDTA‐2506 isolates clustered with BJEU06‐1‐like isolated. Each virus was showed by virus name, isolation year, country, and GenBank accession number. The subgroups of PRRSV‐1 isolates and the four subtypes of PRRSV‐2 isolates were shown in different colors. Both CH‐JX‐2504 and CH‐SDTA‐2506 isolates were marked with red triangles (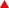).

**Figure:**
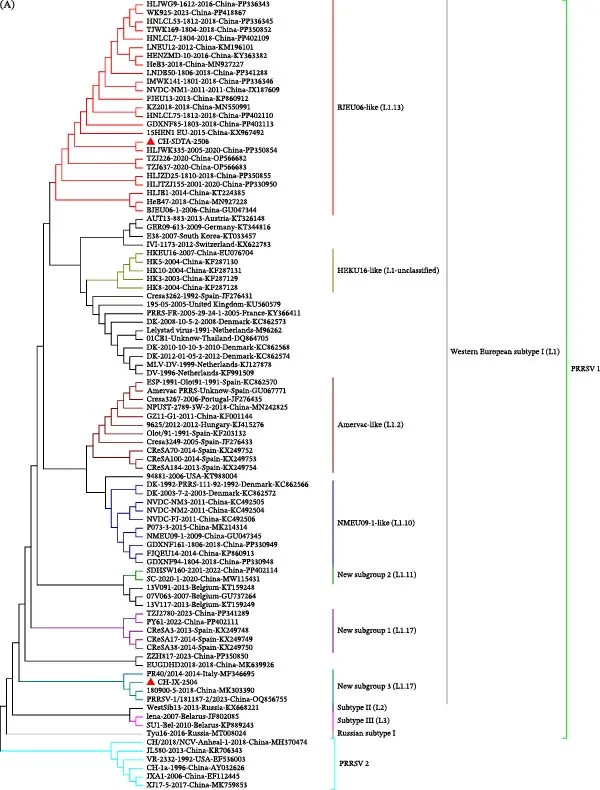

**Figure:**
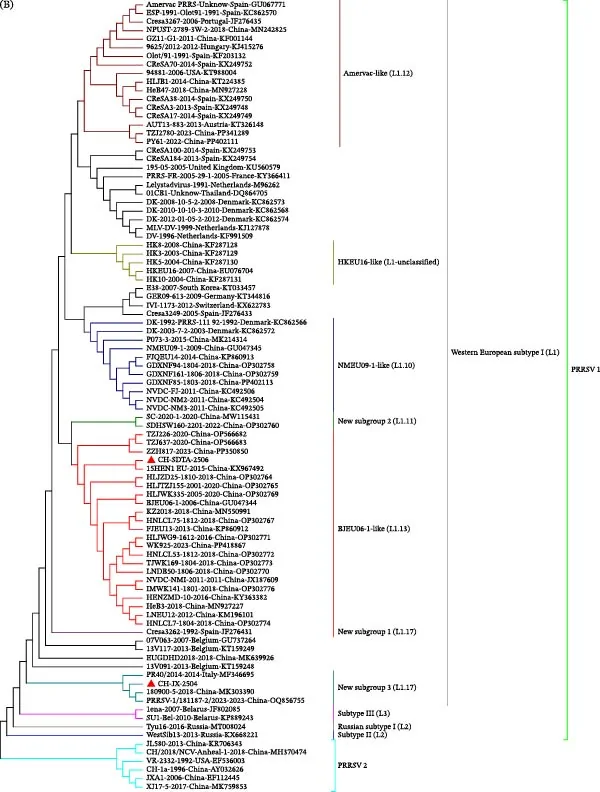

**Figure 5:**
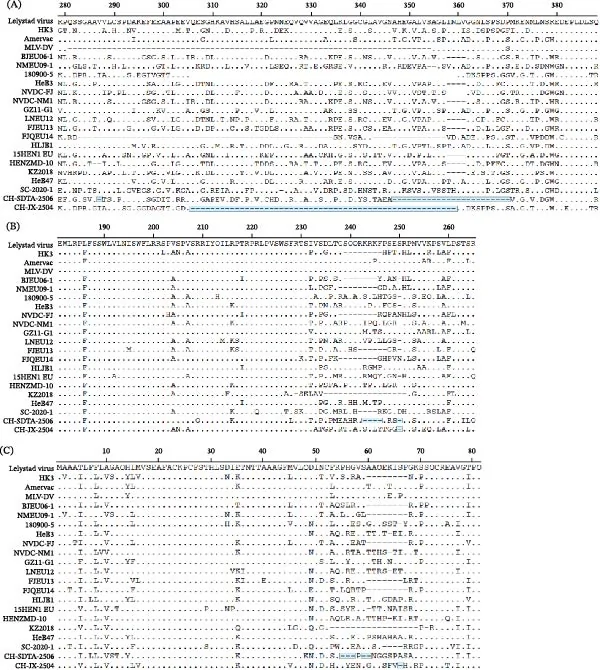
Sequences alignment of partial Nsp2 amino acid and GP3‐GP4 overlapping regions among CH‐JX‐2504, CH‐SDTA‐2506, and representative Chinese PRRSV‐1 strains. (A) Amino acid alignment of partial Nsp2. A consecutive 54 amino acid deletion occurs in the Nsp2 of CH‐JX‐2504, which is identical to 180900‐5 isolate, whereas has (1 + 24) discontinuous amino acid deletion in the Nsp2 of CH‐SDTA‐2506 is highlighted in cyan, suggesting the generation of a new pattern of deletion. (B) Amino acid alignment of partial GP3. CH‐JX‐2504 and CH‐SDTA‐2506 isolates had 1 amino acid deletion and 5 amino acid deletions, respectively in the GP3 is highlighted in cyan. (C) Amino acid alignment of partial GP4. CH‐JX‐2504 and CH‐SDTA‐2506 isolates had 1 amino acid deletion and 5 amino acid deletions, respectively in the GP4 is highlighted in cyan.

To elucidate amino acid insertions and deletions (indels) within Nsp2, GP3, and GP4 of CH‐JX‐2504 and CH‐SDTA‐2506, we aligned the amino acid sequences of these three proteins against reference sequences from Lelystad virus and other PRRSV‐1 strains. The 54 amino acid deletion in Nsp2 (positions 306–359) of CH‐JX‐2504, as well as the 1 amino acid deletion at position 250 in GP3 and position 66 in GP4, are fully consistent with the corresponding deletion patterns of isolate 180900‐5 (Figure 5A–C). In contrast, CH‐SDTA‐2506 has (1 + 24) discontinuous amino acid deletion in Nsp2 (positions 287, 347–370; Figure 5A), and it does not exhibit the classic 5 (4 + 1) discontinuous amino acid deletions of BJEU06‐1‐like PRRSV‐1 in the Nsp2 gene. Sequence alignment of GP3 and GP4 further revealed that CH‐SDTA‐2506 contained unique 5 amino acid deletion in both GP3 (positions 243–246, 250) and GP4 (positions 55–57, 59–60) (Figure 5B,C), which differs from the deletion patterns of other PRRSV‐1 isolates. This suggests that a novel deletion pattern has emerged in BJEU06‐1‐like PRRSV‐1 strains. Thus, these findings highlight the need for strengthened epidemiological surveillance of PRRSV‐1 in China, particularly for BJEU06‐1‐like strains.

### 3.3. Pathogenicity Analysis

**Figure 6:**
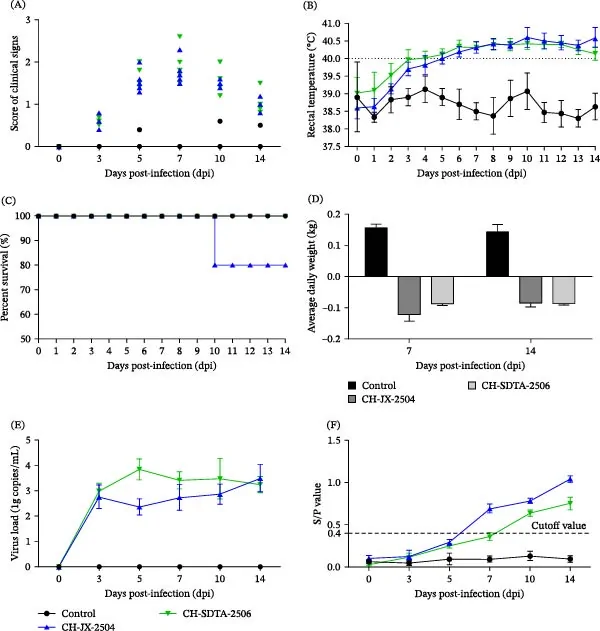
Dynamics of clinical symptom, rectal temperature, survival rate, weight gain, viremia, and antibody level during the challenge study. (A) Clinical scores. (B) Rectal temperature (fever defined as > 40.0°C). (C) Mortality rates of piglets challenged with CH‐JX‐2504 or CH‐SDTA‐2506. (D) Body weight changes during the challenge study. (E) Dynamics of viremia in serum were detected by real‐time RT‐PCR. (F) Serum N protein antibody titers measured by ELISA (s/p > 0.4 indicates seroconversion).

To evaluate the pathogenicity of the CH‐JX‐2504 and CH‐SDTA‐2506 isolates, a pig challenge study was conducted. Challenged piglets exhibited obvious clinical symptoms, including cough, anorexia, diarrhea, and lethargy compared with controls (Figure 6A). Piglets challenged with CH‐SDTA‐2506 developed persistent high fever (>40°C) from 3 to 14 dpi, with the peak temperature reaching 40.6°C at 8 dpi. In contrast, those challenged with CH‐JX‐2504 developed a high fever (>40°C) from 4 to 14 dpi, peaking at 40.8°C at 10 dpi (Figure 6B). One pig challenged with CH‐JX‐2504 died at 10 dpi, while all piglets in the CH‐SDTA‐2506 and control group survived until the end of the study (Figure 6C). Both challenged groups showed significantly reduced average daily weight gain compared with the control group (*p* < 0.05; Figure 6D). Viremia and PRRSV‐specific antibodies were detectable in all challenged piglets. Notably, viral loads in the CH‐SDTA‐2506 group were significantly higher than those in the CH‐JX‐2504 group at 5 dpi (Figure 6E). In addition, PRRSV‐specific antibodies could be detected in all challenged piglets starting from 10 dpi (Figure 6F).

**Figure 7:**
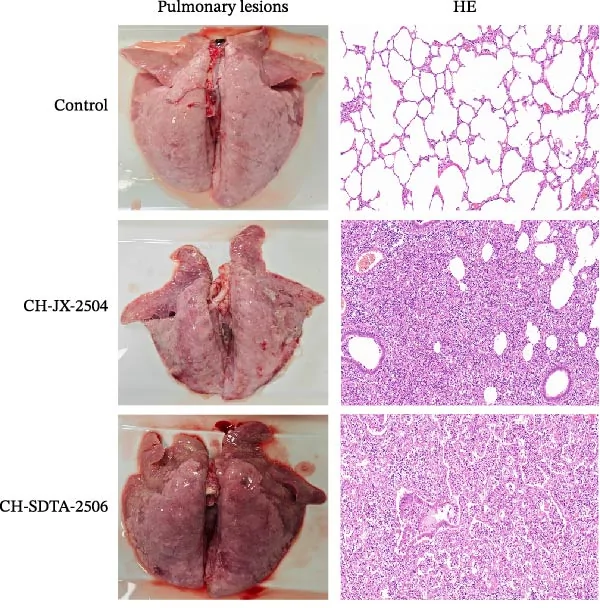
Lung gross lesion and histopathological examination. Pulmonary congestion and hemorrhage were observed in pigs challenged with CH‐JX‐2504 or CH‐SDTA‐2506, but not in control pigs. H&E results revealed that the lungs of pigs challenged with CH‐JX‐2504 and CH‐SDTA‐2506 exhibited increased inflammatory cells and thickening of the alveolar wall but not in control pigs.

After the necropsy of the experimental pigs, lung tissue atrophy, hemorrhage, and pulmonary consolidation were observed in all challenged pigs but not in the control pigs. The histological analysis revealed that the lungs of pigs challenged with these two isolates exhibited marked inflammatory cell infiltration and thickening of the alveolar wall, whereas no significant abnormalities were observed in the control group (Figure 7). These results indicate that CH‐JX‐2504 and CH‐SDTA‐2506 are pathogenic PRRSV‐1 isolates in piglets.

### 3.4. The PRRSV Infection Affects the Structure of the Intestinal Microbiota in Piglets

**Figure 8:**
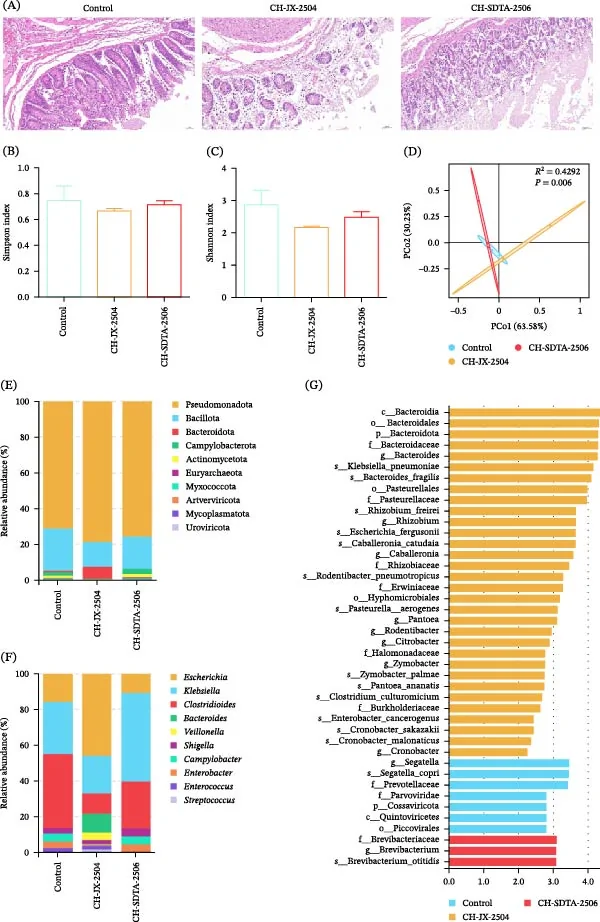
Comparative study of the metagenomic microbiota differences in the jejunum between the two challenged groups and the control group. (A) Representative histological images of H&E‐stained jejunum at a magnification of 20×. (B,C) Alpha‐diversity indices: Simpson’s diversity index (B) and Shannon diversity index (C). (D) Beta‐diversity of gut microbiota shown by principal coordinate analysis (PCoA) using Bray–Curtis distance. (E) Relative abundance of gut microbiota at the phylum level. (F) Relative abundance of gut microbiota at the genus level. (G) Linear discriminant analysis effect size (LEfSe) revealing differentially abundant microbial species.

Jejunum samples were collected from pigs in the control group, CH‐JX‐2504, and CH‐SDTA‐2506 group for histopathological evaluations. Hematoxylin and eosin (H&E) staining revealed that the villi in the control group were well‐distended from the base to the tip, while the tip villi were broken in the CH‐JX‐2504 and CH‐SDTA‐2506 group. Furthermore, compared with the control group, the number of inflammatory cells infiltrating the lamina propria of the jejunum was significantly greater in the two challenge groups (Figure 8A).

The gut microbiota was analyzed using metagenomic sequencing. Three intestinal samples were selected from each group (the control group, CH‐JX‐2504 group, and CH‐SDTA‐2506 group) for analysis. Alpha‐diversity analysis revealed that both the Simpson and Shannon indexes were decreased in the two challenged groups (Figure 8B,C). Beta‐diversity analysis revealed a clear separation between control samples and those from the challenged groups (Figure 8D), indicating significant alterations in the jejunal microbiota composition of PRRSV‐infected piglets.

Analysis of the intestinal microbiota of challenged pigs at the phylum and genus levels gives a clear understanding of changes in intestinal microbiota composition. Pseudomonadota and Bacillota were the most dominant bacteria in all samples (Figure 8E). Further analysis of relative abundance was conducted at the genus level (Figure 8F). Compared with the control group, the relative abundances of *Escherichia*, *Bacteroides*, and *Veillonella* were increased in the CH‐JX‐2504 group, whereas there was no significant change in the CH‐SDTA‐2506 group. Moreover, compared with the control group, the CH‐JX‐2504 group exhibited reduced relative abundance of *Clostridioides*. The above results indicate that the CH‐JX‐2504 group changed the abundance of intestinal microbiota.

Further species‐level linear discriminant analysis effect size (LEfSe) analysis revealed that the CH‐JX‐2504 challenge group was significantly enriched in the class Bacteroidia, order Bacteroidales, phylum Bacteroidota, family Bacteroidaceae, and genus *Bacteroides*. For the CH‐SDTA‐2506 challenge group were specifically enriched in species related to the family *Brevibacterium*. In the control group were significantly enriched in the genus *Segatella* (Figure 8G).

## 4. Discussion

PRRSV‐1 was introduced into China over two decades ago [18]. In recent years, its prevalence and genetic diversity have been continuously increasing, posing substantial challenges to the existing prevention and control strategies [13, 19]. While PRRSV‐2 remains the predominant genotype circulating in Chinese swine herds, the infection rate of PRRSV‐1 has shown a gradual upward trend. PRRSV‐1 has been detected in more than 20 provinces across China, indicating a progressive expansion of its geographical distribution [20]. In this study, two novel PRRSV‐1 strains, CH‐JX‐2504 and CH‐SDTA‐2506, were isolated, and their genomic characteristics, phylogenetic relationships, recombination patterns, and pathogenicity in piglets were systematically characterized. These results further enrich the epidemiological data of PRRSV‐1 in China and confirm the continuous circulation and evolution of pathogenic PRRSV‐1 in domestic swine populations, thereby providing a basis for targeted surveillance and vaccine improvement.

Nsp2 exhibits the highest variability among the Nsps of PRRSV [8]. The ORF3/4 overlapping region represents another hypervariable region besides Nsp2, and deletions in this region are commonly identified in PRRSV‐1 isolates [21–23]. In 2011, Chen et al. [9] first reported mutations in Nsp2 and GP3 of Chinese PRRSV‐1 isolates, and such deletions are considered potential biological markers for the variation of Chinese PRRSV‐1 strains [9]. Amino acid sequence alignment showed that CH‐JX‐2504 had a 54 amino acid deletion in Nsp2 and a 1 amino acid deletion in both GP3 and GP4, with an identical deletion pattern to isolate 180900‐5. It is worth noting that CH‐SDTA‐2506 has (1 + 24) discontinuous amino acid deletion in Nsp2, and it does not exhibit the classic 5 (4 + 1) discontinuous amino acid deletions of BJEU06‐1‐like PRRSV‐1 in the Nsp2, indicating ongoing adaptive evolution within this dominant lineage. Moreover, alignment analysis of GP3 and GP4 revealed that CH‐SDTA‐2506 has a 5 amino acid deletion in both proteins, which differs from the deletion patterns of other PRRSV‐1 isolates, suggesting the emergence of a novel deletion pattern in BJEU06‐1‐like PRRSV‐1 strains. Given that Nsp2 is associated with viral replication and immune evasion, and GP3 and GP4 are involved in receptor binding and neutralizing antibody induction, these indel events may alter viral antigenicity and cellular tropism, regulate viral virulence and host adaptation, and enable the virus to evade immune pressure induced by current PRRSV‐2 vaccines [20, 24]. The identification of the two isolates further confirms the increasing complexity of the PRRSV‐1 population in China and highlights the necessity of long‐term genomic surveillance of PRRSV.

Recombination is a major driving force for the genetic diversity and pathogenic evolution of PRRSV. Extensive recombination events have been identified among domestic PRRSV‐1 strains in China, predominantly originating from BJEU06‐1‐like and NMEU09‐1‐like strains [8, 19]. Recombination may alter viral pathogenicity and facilitate the emergence of virulence‐enhanced strains [11, 25]. High‐throughput sequencing technology has confirmed that coinfection with distinct PRRSV‐1 strains in individual pigs provides an essential precondition for intra‐ and inter‐lineage recombination, thereby facilitating the generation of novel recombinant strains [26]. Typical recombinant isolates including HLJB1 and GD18‐2 display distinct pathogenic characteristics, and vaccine‐induced immune pressure is considered a potential contributor to the emergence of these novel recombinants [20, 24]. Notably, although CH‐JX‐2504 and CH‐SDTA‐2506 were not classified as recombinants in this study, the co‐circulation of multiple PRRSV strains within pig farms and frequent viral transmission between farms substantially increase the risk of subsequent recombination, which may lead to the emergence of genetically complex recombinant variants [26]. To date, the direct causal relationship between recombination and increased virulence has not been fully elucidated. Nevertheless, ongoing recombination continuously elevates the genomic heterogeneity of PRRSV, impairs the cross‐protective efficacy of current commercial vaccines, and poses a substantial obstacle to molecular epidemiological surveillance and comprehensive prevention and control of PRRSV in China.

The pathogenicity experiment results showed that CH‐JX‐2504 and CH‐SDTA‐2506 are both pathogenic to piglets, which can cause clinical symptoms such as high fever, cough, anorexia, and diarrhea, as well as lung lesions, damage the villus structure of the piglet jejunum, and alter the composition of the intestinal microbiota. Notably, the CH‐JX‐2504 has relatively stronger virulence and can lead to piglet death. Although Chinese subtype 1 PRRSV‐1 isolates are generally less pathogenic than HP‐PRRSV‐2 isolates [20], highly pathogenic subtype 1 PRRSV‐1 variants have been reported abroad, such as the Belgium 13V091 and Italian PR40/2014 isolates [27, 28]. Combined with the results of this study that the isolated strain CH‐JX‐2504 could cause piglet death, suggests that domestic PRRSV‐1 strains possess a potential risk of virulence enhancement. It is worth noting that the novel PRRSV‐1 strain GD18‐2 isolated from southern China can vertically spread in pregnant sows and cause severe reproductive disorders [24]. Whether CH‐JX‐2504 and CH‐SDTA‐2506 have similar reproductive pathogenicity and vertical transmission ability still needs further verification, and the relevant research results can provide important references for the health management of sow herds. At present, there is no commercialized PRRSV‐1‐specific vaccine in the Chinese mainland, and existing PRRSV‐2 vaccines fail to provide effective cross‐protection against the emerging PRRSV‐1 strains [24]. Research has shown that PRRSV‐1 can break through the immune barrier established by the PRRSV‐2 vaccination. Moreover, the antiserum induced by a single PRRSV‐1 strain cannot efficiently neutralize other variant strains, indicating that the novel PRRSV‐1 variant has developed a strong immune evasion capacity [20, 24]. Therefore, it is crucial to strengthen the molecular epidemiological monitoring and clarify the genetic evolution and pathogenicity of highly diverse PRRSV‐1 isolates in swine herds.

In conclusion, this study reports the isolation and comprehensive characterization of two novel PRRSV‐1 strains (CH‐JX‐2504 and CH‐SDTA‐2506). Among them, CH‐SDTA‐2506 exhibits distinct genomic features compared with previously identified strains and displays novel deletion patterns in Nsp2, GP3, and GP4. The animal challenge study demonstrated that both strains are pathogenic to piglets. Although one piglet from the CH‐JX‐2504 group died at 10 dpi, infection with both strains caused intestinal villus damage and disruption of the intestinal microbiota structure. Collectively, these findings indicate that the genetic diversity of PRRSV‐1 in the Chinese pig population continues to increase, is constantly evolving, and is widely spreading. It is crucial to conduct long‐term dynamic monitoring of PRRSV‐1 and strengthen the regular monitoring of adult pig herds [29].

## Author Contributions

Yulin Xu, Qihang Xin, Kexin Li, and Jianda Li conceived and designed the experiments. Yulin Xu, Qihang Xin, and Kexin Li performed the experiments. Yulin Xu, Qihang Xin, Kexin Li, Luogang Ding, Yuyu Zhang, Hao Zeng, Fei Liu, Zhi Chen, and Wenbo Sun performed the analysis. Yulin Xu drafted the manuscript. Jiang Yu and Jiaqiang Wu substantively revised this manuscript.

## Funding

This work was supported by the Natural Science Foundation of Shandong Province (Grants ZR2026MS0464, ZR2024QC023), the Natural Science Funds of China (Grant 32402881), the Shandong Province Pig Industrial Technology System (Grant SDAIT‐08), the New High Schools 20 in Jinan of Shandong Province (Grant 202333065), the Taishan Scholars Program, and Agricultural Science and Technology Innovation Project of Shandong Academy of Agricultural Sciences (Grant CXGC2025F21‐2‐1).

## Disclosure

All authors contributed to the article and approved the submitted version.

## Ethics Statement

The animal experiment was approved by the Animal Welfare and Ethics Committee of the Institute of Animal Science and Veterinary Medicine, Shandong Academy of Agricultural Sciences, with the Reference Number of IASVM‐2025‐024.

## Conflicts of Interest

The authors declare no conflicts of interest.

## Supporting Information

Additional supporting information can be found online in the Supporting Information section.

## Supporting information


**Supporting Information 1** Table S1: Metagenomics sequencing quality of CH‐JX‐2504.

**Supporting Information 2** Table S2: Metagenomics sequencing quality of CH‐SDTA‐2506.

## Data Availability

The data that supports the findings of this study are available in the Supporting Information of this article.
