# Supplementary material for: Genetic and Pathogenic Characteristics of Novel PRRSV‐1 Strain CH‐JX‐2504 and CH‐SDTA‐2506 in China
Source: Transbound Emerg Dis. 2026 Aug 24;2026:4674029. doi: 10.1155/tbed/4674029 (PMC13501167; doi:10.1155/tbed/4674029)
Supplement: Supplementary file 1 [file TBED-2026-4674029-s001.docx]

Table S1. Metagenomics sequencing quality of CH-JX-2504.

| QC | Before filtering | After filtering |
| --- | --- | --- |
| Total_reads | 7610790 | 7476072 |
| Total_bases | 1141618500 | 1110948554 |
| Q20_bases | 1113652697 | 1092024544 |
| Q30_bases | 1043573104 | 1029433023 |
| Q20_rate | 97.55% | 98.30% |
| Q30_rate | 91.41% | 92.66% |
