# Supplementary material for: Genetic and Pathogenic Characteristics of Novel PRRSV‐1 Strain CH‐JX‐2504 and CH‐SDTA‐2506 in China
Source: Transbound Emerg Dis. 2026 Aug 24;2026:4674029. doi: 10.1155/tbed/4674029 (PMC13501167; doi:10.1155/tbed/4674029)
Supplement: Supplementary file 2 [file TBED-2026-4674029-s002.docx]

Table S2. Metagenomics sequencing quality of CH-SDTA-2506.

| QC | Before filtering | After filtering |
| --- | --- | --- |
| Total_reads | 9754360 | 9584488 |
| Total_bases | 1463154000 | 1349568972 |
| Q20_bases | 1426332139 | 1332326103 |
| Q30_bases | 1345547443 | 1271887828 |
| Q20_rate | 97.48% | 98.72% |
| Q30_rate | 91.96% | 94.24% |
